# Resistance to glucose starvation as metabolic trait of platinum-resistant human epithelial ovarian cancer cells

**DOI:** 10.18632/oncotarget.14118

**Published:** 2016-12-23

**Authors:** Anna Pastò, Anna Pagotto, Giorgia Pilotto, Angela De Paoli, Gian Luca De Salvo, Alessandra Baldoni, Maria Ornella Nicoletto, Francesca Ricci, Giovanna Damia, Chiara Bellio, Stefano Indraccolo, Alberto Amadori

**Affiliations:** ^1^ Department of Surgery, Oncology and Gastroenterology, University of Padova, Padova, Italy; ^2^ Istituto Oncologico Veneto IRCCS, Padova, Italy; ^3^ Laboratory of Molecular Pharmacology, Oncology Department, IRCCS-Istituto di Ricerche Farmacologiche Mario Negri, Milan, Italy

**Keywords:** glucose addiction, platinum resistance, ovarian cancer, metabolism, autophagy

## Abstract

Deregulated glucose metabolism is observed in cancer but whether this metabolic trait influences response to or is modulated by cytotoxic drugs is unknown. We show here that tumor cells from epithelial ovarian cancer (EOC) patients can be categorized, according to their *in vitro* viability under glucose starvation, into glucose deprivation-sensitive (glucose-addicted, GA) and glucose deprivation-resistant (glucose non-addicted, GNA). When EOC cells were cultured in the absence of glucose, all samples from platinum (PLT)-sensitive patients felt into the GA group; they disclosed higher expression of glucose metabolism enzymes, higher proliferation rates and *in vitro* sensitivity to PLT. Moreover, GA patients showed reduced multi-drug resistance pump expression and autophagy, compared to GNA samples. The close association between PLT sensitivity and glucose metabolic profile was confirmed in a xenograft model, where a stringent parallelism between PLT sensitivity/resistance and glucose metabolism was identified. Finally, in a cohort of naïve EOC patients categorized as GA or GNA at diagnosis, Kaplan Meier curves showed that the GA phenotype was associated with significantly better progression-free survival, compared to GNA patients.

## INTRODUCTION

Epithelial ovarian cancer (EOC) is the most common cause of death in women with gynecologic malignancies [1]. To date, EOC is mostly treated via surgical resection, followed by adjuvant platinum (PLT)- and taxane-based chemotherapy [2]. Nonetheless, about 80% of the patients diagnosed with EOC will relapse after first-line chemotherapy due to drug resistance development [3]. According to the definition of the International Federation of Gynecology & Obstetrics (FIGO), based on the time elapsed between first-line PLT treatment completion and recurrence, EOC patients can be subdivided into different categories [4]. Patients whose disease recurs later than 6 months after cessation of first-line therapy are classified as PLT-sensitive, whereas patients who progress within 6 months are categorized as PLT-resistant. Recurrence is usually accompanied by formation of ascites, a pathologic accumulation of peritoneal fluid containing inflammatory and disseminated tumor cells [5].

The metabolic properties of tumors differ remarkably from those of the tissues of origin. In fact, tumor cells exhibit altered pathways of biomass and energy production which allow them to sustain higher proliferative rates and resist cell death signals, such as those induced by chemotherapeutic agents [6]. Among the major metabolic alterations of cancer cells is the so-called Warburg effect, consisting in enhanced glycolysis under aerobic conditions and increased glucose uptake via over-expression of glucose transporters [7]. Cancer cells preferentially use glycolysis for energy production rather than oxidative phosphorylation; however, whether tumor cell metabolism could be affected by cytotoxic therapy has not been carefully investigated so far. Here we demonstrate that EOC cells exhibit heterogeneous glucose addiction and metabolic profiles; these features strictly correlate with patients’ response to carboplatin and can in some instances be affected by platinum-based therapy.

## RESULTS

### *In vitro* glucose starvation discloses two distinct metabolic phenotypes of EOC cells

**Figure 1:**
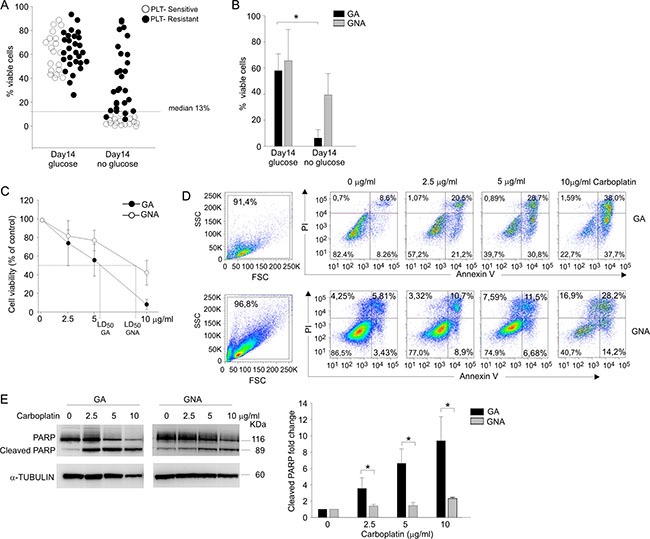
Glucose addiction discriminates two EOC patient categories and correlates with sensitivity to in vitro PLT-treatment (**A**) Flow cytometry analysis of cell viability in ascitic effusion cells isolated from 47 EOC patients categorized as PLT-sensitive/ resistant on the basis of their clinical response to PLT treatment. Cell viability of CD45^neg^/CD44^pos^ tumor cells was recorded after 14-day *in vitro* culture in the presence (glucose) and in the absence (no glucose) of glucose by Live/Dead staining. Each circle represents an individual patient, and the dotted line indicates the median value (13.0%), chosen as cut-off value. (**B**) The bar graph summarizes mean values (± SD) of tumor cells from EOC patients categorized as glucose addicted (GA) or glucose non-addicted (GNA) according to their cell viability after 14 d under glucose starvation. *P < 0.05. (**C**) Cell viability analysis of AnnexinV/PI staining in CD45^neg^/CD44^pos^ tumor cells from GA (*n* = 6) and GNA (*n* = 6) patients treated *in vitro* for 72 h with different doses of carboplatin (2.5, 5 and 10 μg/ml). Data were expressed as mean percent viability (± SD) compared to control. The LD50 value for GA and GNA samples is indicated on the abscissa axis. (**D**) Flow cytometry analysis of cell morphology and viability by AnnexinV/PI staining in CD45^neg^/CD44^pos^ tumor cells from two representative GA and GNA patients, treated *in vitro* for 72 h with different carboplatin concentrations. (**E**) WB analysis of total and cleaved PARP in FACS-sorted CD45^neg^/CD44^pos^ tumor cells of GA and GNA patients, treated *in vitro* for 72 h with different doses of carboplatin. One representative blot is shown on the left panel; the right histogram depicts mean values (± SD) of 6 GA vs 6 GNA patients. Signal intensities of the PARP bands were normalized against the α-tubulin signal. Expression ratios were calculated by dividing normalized signal intensity values obtained for untreated GA or GNA cells. *P < 0.05.

To investigate a possible association between response to chemotherapy and metabolic features of tumor cells, we collected EOC ascitic effusions from 47 carboplatin-treated patients (Table 1) who were categorized as clinically PLT-sensitive or -resistant according to FIGO classification. Tumor cells isolated from patients’ ascitic fluids were cultured *in vitro* either in the presence or in the absence of glucose for two weeks. Interestingly, while under normal culture conditions the viability of tumor cells from PLT-sensitive and resistant patients was comparable (Figure 1A), in PLT-sensitive samples cell viability dropped dramatically upon glucose deprivation, and it was in all cases below the median value calculated for all samples. Instead, PLT-resistant samples collectively displayed lower sensitivity to glucose starvation, although heterogeneous responses were recorded (Figure 1A). Thus, we arbitrarily chose median viability under glucose starvation (13.0%) as a cut-off value to discern two groups, which possibly reflected different states of glucose addiction: glucose deprivation-sensitive (glucose addicted, GA) patients (14-d viability < 13.0%), and glucose deprivation-resistant (glucose non-addicted, GNA) patients (14-d viability ≥ 13.0%) (Figure 1B and Table 1).

**Table 1:** Clinical characteristics of EOC-bearing patients and association between *in vitro* glucose addiction of tumor cells and clinical PLT response

	Platinum-sensitive^a^ *N* = 19(%)	Platinum-resistant *N* = 28(%)	Total *N* = 47 (%)	*p* value^b^
**Histotype**				NS
Endometrioid	1 (5.2)	5 (17.8)	6 (12.71)	
Serous	16 (84.2)	21 (75)	37 (78.7)	
Other (mucinous/clear cells)	2 (10.5)	2 (7.1)	4 (8.5)	
**Stage**				NS
3A	4 (21.0)	3 (10.7)	7 (13.85)	
3B	0 (0.0)	1 (3.6)	1 (4.62)	
3C	12 (63.1)	19 (67.8)	31 (67.69)	
4	3 (15.78)	5 (17.8)	8 (17.02)	
**Grading**				NS
G1	3 (15.7)	2 (7.1)	5 (10.6)	
G2	2 (10.5)	6 (21.4)	8 (17.02)	
G3	14 (73.7)	20 (71.4)	34 (72.3)	
**Age (years)**				
Mean ± SD	64.1 ± 11	64.2 ± 9.40	64.15 ± 10.02	
**GA/GNA status^c^**				*P* < 0.001
GA < 13.0%	19	3	22	
GNA ≥ 13.0%	0	25	25	

a) EOC patients were categorized as PLT-sensitive or PLT-resistant based on time to disease recurrence after platinum therapy completion (FIGO classification): patients who relapsed within 6 months were classified as PLT-resistant, whereas patients relapsing after 6 months were classified as PLT-sensitive.

b) χ^2^ test, NS not significant.

c) EOC patients were categorized as GA or GNA at diagnosis, based on the percent viability of their tumor cells under 14-day *in vitro* glucose starvation.

To experimentally validate the apparent correlation between *in vitro* glucose addiction of EOC cells and clinical response to platinum salts we compared *in vitro* cell viability, after 72 h of carboplatin treatment, of tumor cells isolated from EOC samples defined as GA (*n* = 6) or GNA (*n* = 6) according to their cell viability under glucose starvation. As shown in Figure 1C, mean lethal dose 50 (LD_50_) was 5.77 ± 1.03 μg/ml for GA patients and 8.9 ± 1.4 μg/ml for GNA samples. These results correlated with AnnexinV/PI staining of GA and GNA samples after treatment with three different concentrations of carboplatin. In fact, in tumor cells from GA patients apoptosis was detected at the lowest carboplatin concentration used (2.5 μg/ml), whereas in GNA samples as much as 10 μg/ml were needed to induce a sizable increase in apoptosis (Figure 1D). To confirm these data, we evaluated by WB the effects of carboplatin on PARP cleavage, a well-known marker of apoptosis [8]. As shown in Figure 1E, while in GA samples the fraction of cleaved PARP increased in a dose-dependent manner, in cells from GNA patients an increase in cleaved PARP was evident only when the maximal dose of carboplatin was used.

Finally, we addressed the apparent *in vitro* correlation between PLT sensitivity and glucose addiction also by *in vivo* experiments in a xenotransplantation model. We have previously shown that EOC ascitic effusion cells can be successfully transplanted into immunodeficient hosts [9]. Interestingly, EOC xenotransplants generated from ascitic effusions of GA patients maintained a high *in vitro* sensitivity to glucose deprivation with a high mortality in the absence of this nutrient, whereas viability of xenotransplants from GNA samples was not affected by glucose starvation (Supplementary Table S1). Furthermore, when RAG-2γ^−/−^ mice subcutaneously injected with xenotransplants from GA patients were treated with a glucose analogue (2-DG) which prevents glucose utilization, we observed a significant delay in tumor growth after a short treatment of only four doses of 2-DG (Supplementary Figure S1A). On the contrary, in mice injected with xenotransplants from a GNA patient, up to eight doses of 2-DG were needed to spot a significant difference in tumor volume between treated and control animals (Supplementary Figure S1B). Similarly, we observed a significant control of tumor growth by carboplatin administration in RAG-2γ^−/−^ mice s.c. injected with tumor cells isolated from GA donors, whereas up to 3 weeks were needed to significantly reduce tumor development in mice injected with tumor cells from GNA patients (Supplementary Figure S1C, S1D).

On the whole, these data indicate that *in vitro* and *in vivo* PLT sensitivity is closely associated in most patients with a metabolic profile oriented towards strict glucose addiction.

### Tumor cells from GA patients show higher glycolytic activity compared to GNA subjects

**Figure 2:**
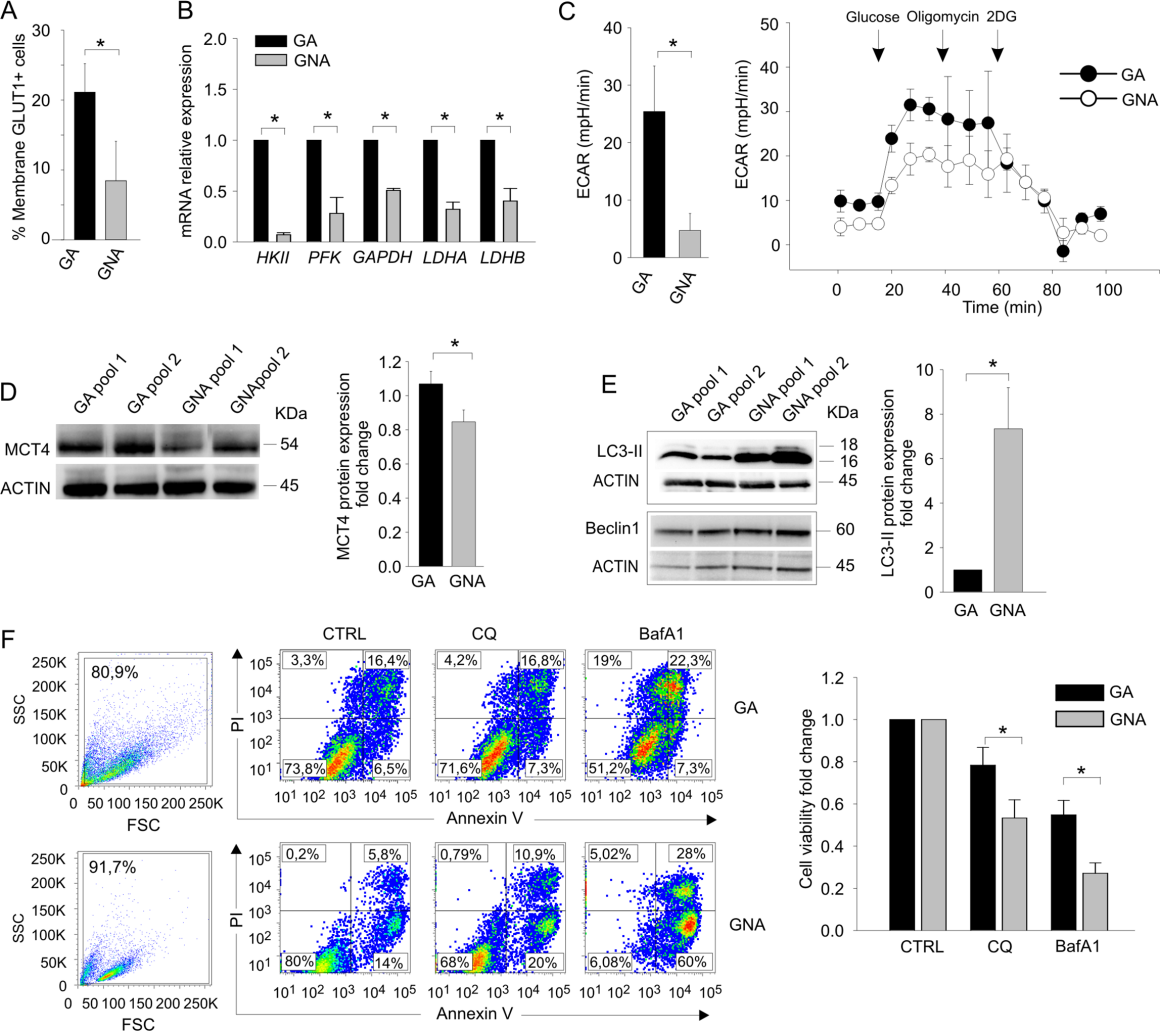
GA and GNA cells exhibit different metabolic profiles (**A**) Flow cytometry evaluation of membrane GLUT1. Data expressed as mean percent values in tumor cells from GA and GNA patients (± SD). *P < 0.05. (**B**) qRT-PCR analysis of key enzymes of the glucose metabolic chain in FACS sorted CD45^neg^/CD44^pos^ tumor cells from GA (*n* = 6) and GNA (*n* = 6) patients. *P < 0.01. (**C**) ECAR analysis in CD45^neg^/CD44^pos^ tumor cells from GA and GNA patients. The left histogram shows the mean values of the basal ECAR in 6 GA vs 6 GNA patients (± SD). *P < 0.05. One representative experiment is shown on the right; the first three points of the graph indicate the basal ECAR ratio in glucose starvation. At different time points (arrows) the indicated inhibitors of OXPHOS (oligomycin) or glycolysis (2DG) were added. (**D**) WB analysis of MCT4 lactate transporter in pooled CD45^neg^/CD44^pos^ tumor cells from GA or GNA patients (pools #1) and xenotransplants (pools #2). Each pool consisted of 3 GA or 3 GNA samples. After normalization against actin, expression ratios were calculated by dividing GNA signal intensity values by those of GA cells. On the left a representative blot, on the right graph depicts mean expression ratios in 8 different pools (± SD). *P < 0.05. (**E**) WB analysis of Beclin1and LC3-II proteins in CD45^neg^/CD44^pos^ tumor cell pools of GA or GNA patients. One representative blot is shown on the left, while the right histogram shows the mean values of 4 different experiments (three samples/pool; ± SD). *P < 0.05. (**F**) Left panel: flow cytometry analysis of cell morphology and viability by AnnexinV/PI staining in two representative GA and GNA samples, treated *in vitro* for 72 h with 20 μM CQ or 100 nM BafA1. Right panel: histogram displaying the mean fold change (± SD) in cell viability, calculated by dividing the percentage of live cells after treatment by untreated values of 4 GA and 5 GNA samples. *P < 0.05.

Since these results could underlie differences in the machinery controlling glycolytic activity between GA and GNA patients, we investigated markers associated with glucose metabolism. First, we evaluated the expression of GLUT1, one of the major glucose transporters [10]. By flow cytometry, we found significantly higher surface expression of GLUT1 in tumor cells from GA (21.13 ± 4.07%) compared to GNA samples (8.44 ± 5.60%, Figure 2A). We next compared mRNA expression levels of several genes encoding for key components involved in glycolysis, such as hexokinase II (*HKII*), phosphofructokinase (*PFK*), glyceraldehyde 3-phosphate dehydrogenase (*GAPDH*) and lactate dehydrogenase (*LDH*). As depicted in Figure 2B, tumor cells isolated from GA patients showed significantly higher expression of all transcripts analysed, compared to GNA samples. These results were supported by Seahorse analysis of extra-cellular acidification rate (ECAR, a surrogate marker of lactic acid production) in short-term *ex vivo* cultures. Tumor cells from GA patients presented significantly higher basal ECAR values compared to GNA samples (Figure 2C, left panel), suggesting higher rate of lactate production. Moreover, when the analysis was performed by adding back glucose to cells initially incubated under glucose starvation, a stronger increase in ECAR was measured in GA compared to GNA samples, and this modulation was completely abrogated by 2-DG (Figure 2C, right panel), indicating that it was due to glycolysis. Accordingly, WB analysis showed a significantly higher expression of MCT4, the major lactate transporter, in tumor cells from GA patients (Figure 2D). On the other hand, oxygen consumption rate (OCR) did not show any significant difference between the two groups (Supplementary Figure S2A). Moreover, treatment with oligomycin dramatically reduced OCR, as expected, but did not increase ECAR in GA and GNA cells, indicating that the two cell subsets have a limited glycolytic reserve (Figure 2C). Similarly, we did not measure significant variation in mRNA expression levels of several key genes involved in mitochondrial activity regulation, including *ATP5B*, *NDUFS2*, *PKM* and PDH, in GA compared to GNA samples (Supplementary Figure S2B).

Altogether, these observations indicate that tumor cells from glucose-addicted EOC patients show higher glycolytic activity, compared to GNA subjects.

### Tumor cells from GNA patients rely more on autophagy compared to GA patients

It has been recently demonstrated that autophagy, a multistep process of intracellular self-digestion [11], is activated in response to cytotoxic drugs [12] and could mediate the acquisition of a chemo-resistant phenotype in some tumor cells. We thus compared the *ex vivo* autophagic activity in GA and GNA patients by Western blotting. Strikingly, tumor cells from GNA patients displayed significantly higher levels of LC3-II, the autophagosome-associated form of the LC3 protein [13], than GA samples, indicating a more active basal autophagy (Figure 2E). In contrast, protein levels of Beclin1, a master regulator of autophagy, were comparable between GA and GNA cells, thus excluding a correlation between GA/GNA phenotype and Beclin1 expression (Figure 2E). The levels of LC3-II were also determined after clamping autophagosome consumption with bafilomycin A1 (BafA1), an inhibitor of autophagy. This allowed us to measure the autophagic flux as the difference in LC3-II protein levels between BafA1-treated and untreated cells. As shown in Supplementary Figure S3A, GA and GNA samples presented a comparable autophagic flux. Similar results were obtained treating the cells with chloroquine (CQ), another inhibitor of autophagy, and labelling them with a fluorescent dye that specifically binds autophagosomes (Supplementary Figure S3B). Finally, to investigate whether autophagy contributed to survival of GNA cells, we treated cells with CQ or BafA1 for 72 h and compared their viability via AnnexinV/PI co-staining. As shown in Figure 2F, GNA samples were more sensitive to this treatment, as they displayed a significantly lower viability than GA samples in the presence of both autophagy inhibitors.

These results indicate that, despite autophagy is equally efficient in terms of flux in GA and GNA samples, the latter have a markedly increased basal activation, which could contribute to their resistance to both platinum treatment and glucose deprivation.

### GNA samples are characterized by low proliferation and high MDR pump expression

**Figure 3:**
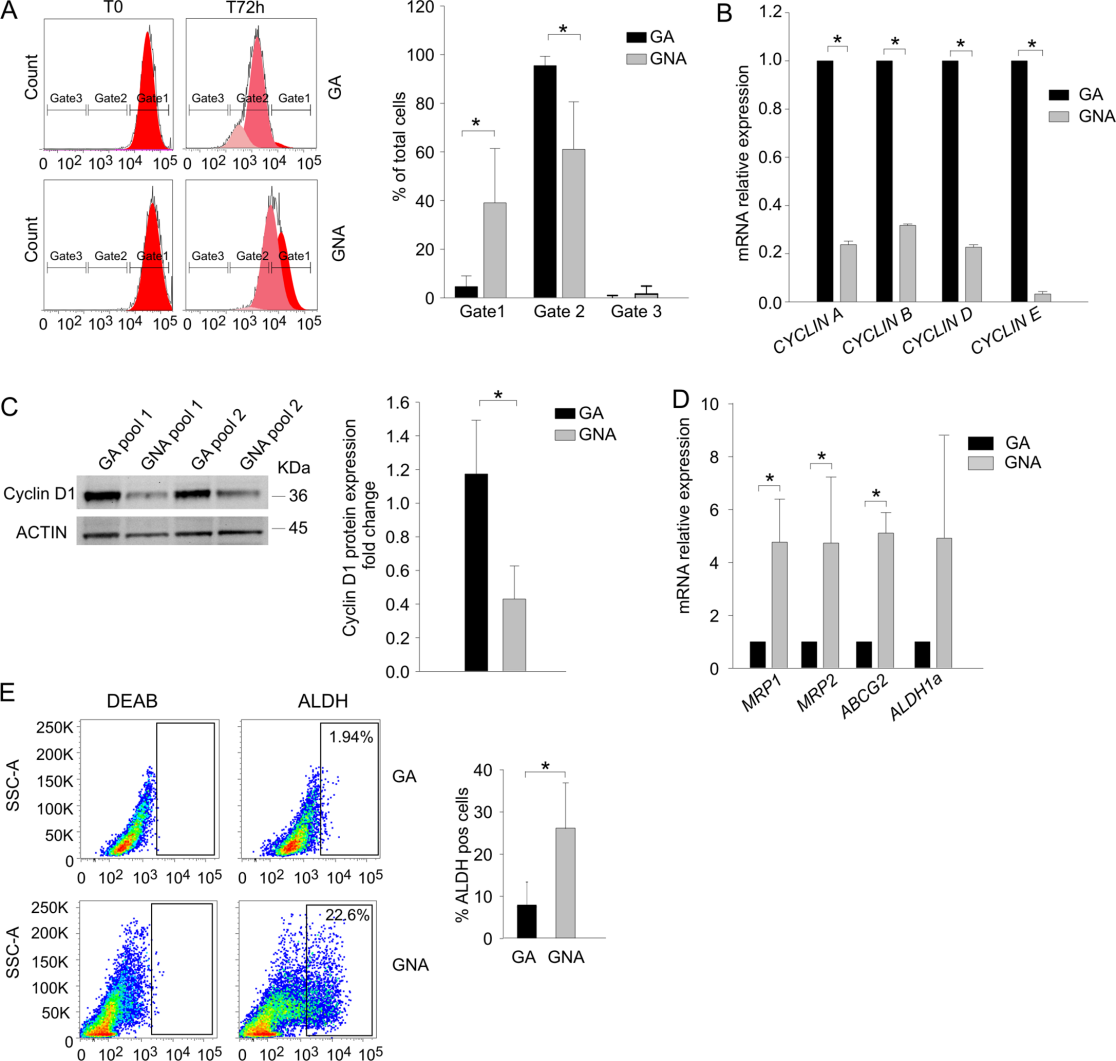
GNA cells present lower proliferation rate and higher MDR pump expression (**A**) The proliferation rate of GA vs. GNA ascitic effusion cells was evaluated by CFSE staining in CD45^neg^/CD44^pos^ tumor cells. Three gates were set according to dye intensity (time 0; left panel). For each gate, the mean percentage of CFSE^+^ cells was quantified 72 h after staining. One representative experiment is shown on the left; mean values (± SD) in 5 GA and 4 GNA patients are plotted on the right. *P < 0.05. (**B**) qRT-PCR analysis of cyclin expression in FACS sorted CD45^neg^/CD44^pos^ tumor cells from EOC ascitic effusions. Data were expressed as mean relative expression values in tumor cells from GA (*n* = 6) compared to GNA (*n* = 6) patients (± SD). *P < 0.001. (**C**) Left panel: Representative WB analysis of Cyclin D expression in pools of CD45^neg^/CD44^pos^ tumor cells from GA and GNA patients (pools #1) and patient-derived xenotransplants (pools #2). Each pool consisted of 3 GA or GNA samples. Right panel: The signal intensity of the Cyclin D bands was normalized against the actin signal; expression ratios were calculated by dividing normalized signal intensity values obtained for GNA cells by those obtained for GA cells. The graph shows mean expression ratios (± SD) in 8 different pools. *P < 0.05. (**D**) qRT-PCR analysis of multidrug resistance (MDR) pump expression in FACS sorted CD45^neg^/CD44^pos^ ascitic effusion cells from GA (*n* = 6) and GNA (*n* = 6) patients. Data were expressed as mean relative expression values (± SD). *P < 0.05. (**E**) *Ex vivo* flow cytometry analysis of ALDH activity in CD45^neg^/CD44^pos^ EOC ascitic effusion cells from GA (*n* = 20) and GNA (*n* = 15) patients. Gates were set on the isotype control, and values indicate the percentage of ALDH^+pos^ cells. One representative experiment is shown on the left; the right histogram shows mean percent values (± SD). *P < 0.05.

These observations prompted us to explore the mechanisms that could explain why platinum-based therapy was more efficient in GA compared to GNA patients. Previous studies indicate that PLT sensitivity relies on two critical parameters: the proliferation rate (which renders tumor cells a more suitable target for the DNA-damaging effects of the drug) and the ability of malignant cells to extrude toxic compounds through multidrug-resistant (MDR) pumps and detoxifying enzymes [14]. We first monitored cell proliferation in EOC primary samples after staining with CFSE, a membrane dye that enables the evaluation of cell division rate by flow cytometry [15]. Three gates were identified at the beginning of the experiment (T0) according to the dye intensity (Figure 3A, left panel); after 72 h *in vitro* culture, the cell percentage in each gate was recorded. As shown in Figure 3A (right panel), cells from GNA patients persisted longer in gate #1 compared to GA cells, indicating a slower proliferation rate. We also addressed expression of Cyclins, whose levels are increased in cycling cells. As shown in Figure 3B, mRNA levels of *CYCLIN A*, *B*, *D* and *E* were significantly higher in tumor cells from GA patients compared to GNA samples. For Cyclin D1, WB analysis fully confirmed these results (Figure 3C). Moreover, we quantified *ex vivo* mRNA expression levels of several MDR pump genes and detoxifying enzyme in tumor cells from GA and GNA patients. As shown in Figure 3D, GNA samples displayed significantly higher expression of three out of four of the genes analyzed. *ALDH1A* mRNA levels did not significantly differ between the two patient categories, mainly due to its heterogeneous expression in GNA samples (Figure 3D); however, when enzymatic activity of ALDH was measured by the ALDEFLUOR^®^ assay, GNA samples showed a 3-fold higher expression of this detoxifying enzyme (Figure 3E).

It has been demonstrated [16] that higher MDR expression and resistance to platinum treatment are key hallmarks of cancer stem cells (CSC). We and others have demonstrated that in ovarian cancer [17] and glioblastoma [18, 19], CSC are characterized by OXPHOS metabolism and resistance to glucose starvation. Hence, we evaluated in GA and GNA samples the percentage of cells co-expressing CD44 and CD117, a recognized marker of EOC CSC [17, 20]. We did not found any difference between GA and GNA patients (data not shown), thus implying that the different phenotype of GA and GNA samples was likely not attributable to their CSC content.

### PLT may favor the GNA phenotype in an EOC-derived xenograft model

**Figure 4:**
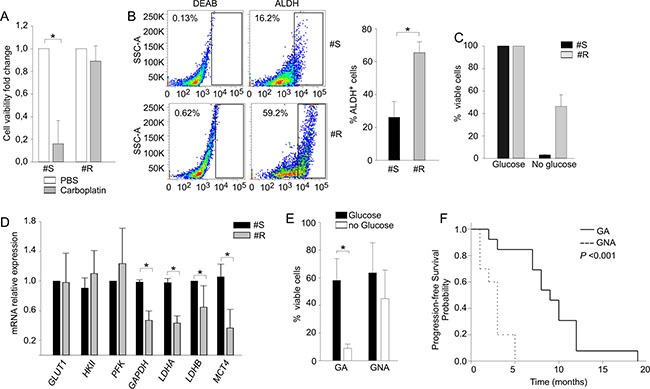
Prolonged PLT-treatment influences the response to glucose starvation in a xenotransplantation model (**A**) Cell viability analysis performed with AnnexinV/PI staining in PLT-sensitive EOC xenografts (#S) and their resistant counterpart (#R) after 72 h of carboplatin treatment. Data are expressed as ratio between treated and untreated counterpart. (**B**) ALDH activity analysis in PLT-sensitive EOC xenografts (#S) and their resistant counterpart (#R) obtained as described in Materials and Methods. On the left one representative sample is shown; the right histogram shows mean values (± SD) of three biological replicates for two different samples. (**C**) Cell viability analysis of PLT-sensitive EOC xenografts (#S) and their resistant counterpart (#R) recorded after 7-day *in vitro* culture in the presence (glucose) and in the absence (no glucose) of glucose by Live/Dead staining. (**D**) qRT-PCR analysis of key enzymes involved in the glucose metabolic chain in PLT-sensitive EOC xenografts (#S) and their resistant counterpart (#R). Data are expressed as mean relative expression values (± SD). *P < 0.05. (**E**) Flow cytometry analysis of cell viability after 14 d in the presence or absence of glucose. The graph summarizes mean values (± SD) of tumor cells isolated from 23 EOC samples of untreated patients categorized as GA or GNA according to their cell viability under glucose starvation. *P < 0.05. (**F**) Kaplan-Meier curve showing the association between the GA/GNA phenotype and progression-free survival in a cohort of untreated EOC patients.

The close association between glucose metabolism and PLT sensitivity was further strengthened in a mouse model, where the platinum-naïve sample was available. To this end, we analyzed parental PLT-sensitive (#S) and their PLT-resistant (#R) derivatives, made resistant by prolonged *in vivo* PLT treatment and selection, as detailed in Materials and Methods. Tumor cells isolated from #S and #R xenotransplants were preliminary treated *in vitro* for 72 h with 20 μg/ml of carboplatin; as expected, a strong reduction of cell viability was measured in #S samples, whereas almost 90% of the cells survived treatment in #R samples (Figure 4A). In addition, when we evaluated ALDH activity at tumor harvest, a three-fold higher expression of this detoxifying enzyme was found in #R samples (mean 65% ± 6.6%), compared to the PLT-sensitive counterpart (#S, mean 21.2 ± 4.8%, Figure 4B). Moreover, when these cells were maintained *in vitro* under glucose deprivation, cell viability was significantly higher in #R compared to #S samples (Figure 4C). In contrast, negligible differences in cell viability of #R and #S cells were detected under glutamine deprivation (data not shown). To better evaluate the metabolic alterations induced by PLT treatment, we also analyzed mRNA expression levels of several genes involved in glucose metabolism, including *GLUT1*, *HKII*, *PFK*, *GAPDH*, *LDHA*, *LDHB* and *MCT4*. As shown in Figure 4D, genes encoding for enzymes that transform pyruvate into lactate and regulate its extrusion outside the cells were substantially downregulated in #R samples.

On the whole, these data confirm the strict parallelism between glucose addiction and PLT sensitivity, and demonstrate that PLT resistance may be associated with a change in glucose metabolic profile.

### The GA/GNA phenotype predicts clinical responsiveness in untreated patients

Besides being selected by chemotherapy, it is possible that the GA/GNA phenotype and glucose addiction could be an intrinsic metabolic feature that some EOC patients display before chemotherapy. To investigate this possibility, we evaluated in a prospective cohort of 23 patients, who did not receive any treatment before sampling, the 14-d viability of their tumor cells in the absence of glucose (Figure 4E). At diagnosis, 13 patients (56.5%) were categorized as GA, and 10 (43.5%) as GNA; all underwent first-line PLT-based chemotherapy, and the time to tumor relapse was recorded over 18 months of follow-up. As shown in Table 2, in this prospective cohort no correlation emerged between PLT-sensitive/resistant phenotypes and tumor stage, grading or histotype. On the other hand, a significant association between the GA/GNA phenotype and clinical sensitivity/resistance to first-line PLT chemotherapy was evidenced (Table 2). In addition, as shown by the Kaplan-Meier curves (Figure 4F), GA patients showed better median progression-free survival (PFS) (9.1 months, 95% CI: 7.0–12.0), compared to GNA patients (2.7 months, 95% CI: 1.0–3.0; *P < 0.001*) over the follow-up period.

**Table 2:** Clinical characteristics of untreated EOC-bearing patients and association between *in vitro* glucose addiction of tumor cells and clinical PLT response

	PLT-sensitive^a^ *N* = 11 (%)	PLT-resistant *N* = 12 (%)	Total *N* = 23 (%)	*p* value^b^
**Histotype**				NS
Endometrioid	2 (18.2)	0 (0)	2 (8)	
Serous	9 (81.8)	7 (58.3)	16 (69)	
Other (mucinous/clear cells)	–	5 (41.6)	5 (21)	
**Stage**				NS
3A–3B	2 (18.2)	2 (16.6)	4 (17.3)	
3C–4	9 (81.8)	10 (83.4)	19 (82.6)	
**Grading**				NS
G1–G2	2 (18.2)	3 (25)	5 (21)	
G3	9 (81.8)	9 (75)	18 (78)	
**Age (years)**				
Mean ± SD	63.3 ± 9.53	74.7 ± 7.1		
**Surgery**				NS
Yes	7 (63.6)	5 (41.6)	10 (43.5)	
No	4 (36.4)	7 (58.3)	13 (56.5)	
**GA/GNA status^c^**				*P* < 0.001
GA < 13.0%	11 (100)	2 (16.6)	13 (56.5)	
GNA ≥ 13.0%	0 (0)	10 (83.4)	10 (43.5)	

a) EOC patients were categorized as PLT-sensitive or PLT-resistant based on time to disease recurrence after first-line platinum therapy; patients who relapsed within 6 months were classified as PLT-resistant, whereas patients relapsing after 6 months were classified as PLT-sensitive (according to FIGO classification).

b) χ^2^ test, NS not significant.

c) EOC patients were categorized as GA or GNA at diagnosis, based on the percent viability of their tumor cells under 14-day *in vitro* glucose starvation (< 13.0% or ≥ 13.0%, respectively).

Altogether, although limited by the low number of patients analyzed, our results seem to indicate that GNA status could predict refractoriness to PLT treatment in naïve EOC patients.

## DISCUSSION

Although platinum resistance is a well-established phenomenon and several mechanisms of resistance have been advanced [21], whether the metabolic properties of tumor cells influence response to or are modulated by cytotoxic drugs is unknown. We investigated these issues in primary samples from ovarian cancer patients and in a xenotransplantation model. Our results clearly identify two distinct metabolic profiles within EOC patients. Based on *in vitro* sensitivity to glucose starvation of tumor cells, patients’ samples could be discriminated into glucose addicted (GA) and glucose non-addicted (GNA). Our data showed a more pronounced Warburg effect in GA samples, which were significantly enriched in PLT-sensitive patients. GA cells presented higher *in vitro* sensitivity to PLT, which could be in part explained by a higher proliferation rate as well as a more active glycolytic machinery, in line with the well-established connections between proliferation and Warburg effect [7]. Nonetheless, the possible contribution to PLT sensitivity of lower MDR pump expression in GA patients (Figure 3D–3E) should also be considered.

These results are partially at odds with previous findings obtained by metabolic characterization of tumor cells selected for platinum-resistance *in vitro*. Indeed, while Jin *et al*. reported that platinum-resistance is associated with down-modulation of glycolysis in ovarian cancer cell lines [22], Catanzaro *et al*. showed that cisplatin-resistant ovarian cancer cells were characterized by reduced mitochondrial activity and higher glucose-dependency, when compared to the cisplatin-sensitive counterpart. These features were associated with increased glucose-uptake and consumption as well as increased expression and enzymatic activity of the pentose phosphate pathway enzyme glucose-6-phosphate dehydrogenase [23]. These contrasting results may depend on the marked differences in the experimental models used in these studies. Our findings have been obtained by analyzing metabolic traits of tumors treated *in vivo* - either in the patients or in xenotranplants - with platinum, whereas Catanzaro *et al*. investigated the adaptive metabolic changes of tumor cells exposed *in vitro* to this drug. This difference could be important, in light of recent publications remarking the impact of the environment on the metabolic phenotype of tumor cells [24]. Along this line, Matassa *et al*. recently observed that oxidative metabolism drives inflammation-induced platinum resistance in ovarian cancer [25]. These results, together with ours, challenge the current assumption that increased glycolysis correlates with poor prognosis, at least in ovarian cancer.

**Figure 5:**
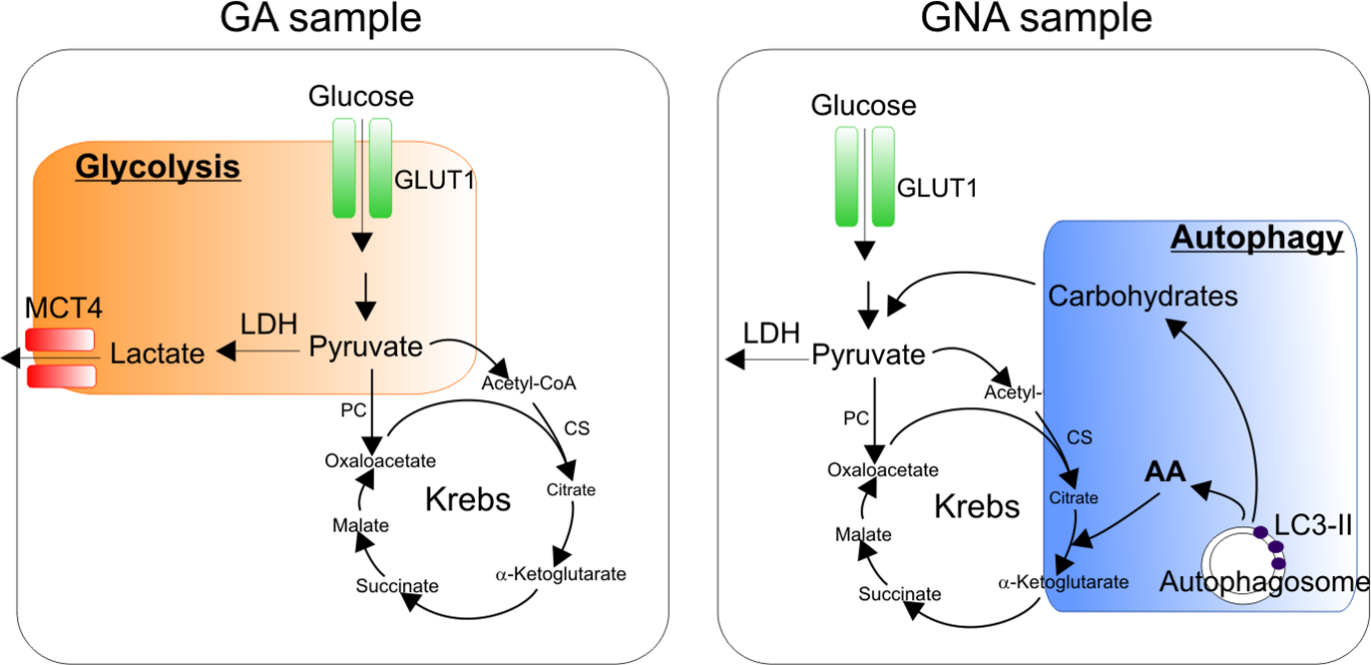
Working hypothesis: dominant metabolic pathways in glucose addicted and glucose non-addicted EOC cells GA samples mostly rely on the Warburg effect with high levels of glucose utilization through glycolysis (orange box). GNA samples, instead, are less dependent on glycolysis, thus implying the utilization of alternative energetic/catabolic pathways such as autophagy (blue box), which degrades damaged cellular organelles in order to recycle their components and produce metabolic intermediates such as carbohydrates or amino acids (AA) that fuel the Krebs cycle.

Intriguingly, the low glycolytic metabolism of GNA samples was counterbalanced by their higher basal autophagic activity and response to autophagy inhibitors than GA cells. Aberrant autophagy represents an emerging hallmark of cancer [26]. This catabolic pathway, mediated by the lysosome compartment, enables cells to degrade damaged cellular organelles in order to recycle their components; therefore, autophagy represents a way for tumor cells to survive the energetic stress they experience in the tumor microenvironment [27]. We speculate that autophagy could be exploited by GNA samples to support their energy needs, since glucose utilization is apparently more dispensable for these cells (Figure 5). Moreover, increased autophagy could also mediate resistance to platinum, as observed in other models [28]. We investigated whether inhibition of autophagy could affect the response to platinum treatment. However, the prominent pro-apoptotic effects of CQ did not enable us to conclude that autophagy blockade could restore platinum sensitivity in GNA cells (data not shown).

Another key finding of our work is that certain metabolic features of tumor cells could be modulated by carboplatin. Although we could not perform metabolic characterization of EOC samples at diagnosis in a large number of patients, serial sampling before and after PLT-treatment revealed a switch from GA to GNA status after platinum treatment in about 1/4 of the cases analyzed (data not shown); in contrast, we never observed a conversion from the GNA to the GA phenotype. The surprising parallelism between PLT - resistance and resistance to glucose starvation was also confirmed in a xenotransplantation model, where we demonstrated that acquisition of a PLT-resistant phenotype and resistance to *in vitro* glucose starvation is accompanied by a dramatic change in the expression of the glucose metabolism-associated machinery (Figure 4D–4E). Currently, it is not clear whether PLT-treatment could in some instances modulate the metabolic profile of tumor cells or rather select a naïve population of less glycolytic cells (GNA phenotype). In the latter scenario, GNA samples would contain a more prominent population of poorly glycolytic cells endowed with primary resistance to PLT compared to GA samples, in which the GNA population could still be present but as a minority. Further studies are warranted to explore more in depth the mechanistic aspects of this issue. In any case, our results are consistent with a previously proposed concept of oncogenic-type of resistance to anti-cancer drugs [29, 30]. In fact, it could well be that oncogenic alterations that render cells resistant to anti-cancer drug are positively selected by chemotherapy and simultaneously make the cells capable of tolerating low glucose conditions.

Finally, we observed that the *in vitro* glucose addiction of primary tumor cells could anticipate the response to first-line PLT-based schedules of the patients from which they were isolated. In fact, the different PLT sensitivity of GA and GNA patients was confirmed *in vivo* in a limited cohort of EOC patients whose samples were collected before any chemotherapic treatment. As shown by Kaplan-Meier analysis, patients categorized at diagnosis as GA according to their glucose addiction and prospectively followed for over 18 months, appeared PLT-sensitive according to the FIGO definition [4], and showed better PFS compared to GNA patients.

In conclusion our study demonstrates that EOC samples can be stratified according to their glucose addiction. In particular, by analyzing tumor cell sensitivity to *in vitro* glucose deprivation we can identify glucose addicted (GA) samples and glucose non-addicted (GNA) ones. Importantly, the GA/GNA status correlates with response to PLT treatment both *in vitro* and *in vivo* and can be affected by prolonged PLT therapy. Even though we are aware of the limited dimension of our patient cohort, our observations suggest that this *in vitro* glucose addiction assay may represent a reliable and novel parameter to predict clinical sensitivity to PLT in ovarian cancer.

## MATERIALS AND METHODS

### EOC primary samples and xenografts

This study was approved by the Institutional Ethics Committee for patient studies. After obtaining written informed consent, 70 patients diagnosed with EOC entered this study: 47 had received chemotherapy prior to sampling, whereas 23 were sampled at diagnosis before any surgery/chemotherapy. Patient's clinical and pathologic features are summarized in Table 1. EOC cells were isolated from ascitic effusion fluids as reported elsewhere [31] and from ascitic effusions generated after the orthotopic injection of EOC cells into immunocompromised mice. All the experiments were performed on gated or FACS-sorted CD45-negative and CD44-positive (CD45^neg^/CD44^pos^) tumor cells to exclude myeloid and lymphoid contamination.

### Glucose deprivation assay and *in vitro* cell treatments

EOC ascitic effusion cells were cultured at 6 × 10^4^ cells/ml in complete or glucose-deprived RPMI medium (Sigma-Aldrich, St. Louis, MO). After 14 days, cell viability of CD45^neg^/CD44^pos^ tumor cells was evaluated by flow cytometry with Live/Dead (Life Technologies, Monza, Italy) staining as described [17].

Carboplatin (2.5, 5 and 10 μg/ml), chloroquine (CQ, 20 μM, Sigma-Aldrich) and bafilomycin A1 (BafA1, 100 nM, Sigma-Aldrich) were added to the complete medium, and cell viability evaluated 72 h later by AnnexinV/PI staining as described [17]. For autophagic flux analysis cells were treated with BafA1 (100 nM) for 2 h or CQ (50 μM) overnight.

### Animal studies

Severe combined immunodeficiency (SCID) and RAG-2γ^−/−^ mice were obtained from internal breeding. Procedures involving animals and their care were performed according to institutional guidelines that comply with national and international laws and policies (EEC Council Directive 86/609, OJ L358, 12 December 1987). Patient derived xenografts (PDX) were generated by injecting intraperitoneally (i.p.) into SCID mice 10^6^ tumor cells from ascitic effusions of EOC patients as reported elsewhere [32]. For carboplatin treatment, 5 × 10^5^ CD45^neg^/CD44^pos^ tumor cells isolated from high-grade serous ovarian cancer xenotransplants (classified as GA or GNA at baseline) were injected subcutaneously (s.c.) in 200 μl of Matrigel in both dorsolateral flanks of RAG-2γ^−/−^ mice. When tumors reached 100 mm^3^ volume, mice were randomized in two groups, and either treated with 4 doses of carboplatin (50 mg/Kg weekly) or with equal saline amounts as a control. Tumor growth was evaluated by caliper measurements. Mice were sacrificed when the tumors of the control group reached 600 mm^3^ volume.

To investigate the possible correlation between PLT response and glucose metabolism, we selected two EOC xenotransplants sensitive to cisplatin treatment (#S), which were injected s.c. into nude mice (Envigo RMS, Corezzana, Italy). Mice were treated with multiple cycles of cisplatin (5 mg/Kg once every 3 weeks/cycle) in order to obtain the cisplatin-resistant counterpart (#R); meanwhile the sensitive counterpart was maintained by serial passage in mice receiving PBS. Experiments were performed after a total of 5–8 cisplatin cycles.

### Flow cytometry (FACS) analysis

FACS sorting was performed with a MoFlo Astrios cell sorter (Coulter Electronics, Brea, CA) after gating CD45^neg^/CD44^pos^ tumor cells with anti-CD45 (1:10, Miltenyi Biotec, Bergisch Gladbach, Germany) and anti-CD44 (1:1000, Abcam , Cambridge, UK) antibodies. For flow cytometry analysis, cells were stained with Live/Dead and anti-CD44 (Abcam), CD45 (Miltenyi), CD117 (1:100, Miltenyi Biotec) and GLUT1 (1:1000, Abcam) antibodies.

Aldehyde dehydrogenase (ALDH) activity was determined by a fluorogenic dye (ALDEFLUOR^®^ assay; Stem Cell Technologies, Vancouver, Canada), as described elsewhere [33]. The specific ALDH inhibitor diethylaminobenzaldehyde (DEAB) was used as a control.

To evaluate the proliferation rate, EOC effusion cells were stained with carboxyfluorescein-diacetate-succinimidylester (CFSE; Life Technologies) as described elsewhere [15], and seeded at 6 × 10^4^ cells/well in RPMI medium. Flow cytometry analysis was performed 72 h later. For autophagic flux analysis, CYTO-ID^®^ Autophagy detection kit (Enzo, New York, NY) was used according to the manufacturer's instructions. All the analyses were performed with FACS LSRII (BD); data were analyzed with Flow Jo (TreeStar, Ashland, OR).

### Seahorse analysis

FACS-sorted CD45^neg^/CD44^pos^ tumor cells were seeded at 2 × 10^4^/well in XF24 V7 Extracellular Flux Analyzer microplates (Seahorse Biosciences, Copenhagen, Denmark) in complete RPMI medium or RPMI without glucose (according to experimental design), and incubated for 4 h at 37°C, 5% CO_2_. The assay was initiated by medium replacing, followed by the addition over a 2 h-analysis of the following reagents: Oligomycin (0.8 μM), FCCP (900 nM), Rotenone (1 μM), Antimycin (1 μM), 2-DG (100 mM) or glucose (10 mM) all from Sigma-Aldrich. Three measurements were done in triplicate for each reagent addition.

### Western blotting (WB)

FACS-sorted CD45^neg^/CD44^pos^ tumor cells were subjected to SDS-polyacrylamide gel electrophoresis and WB as reported [17]. The membranes were hybridized using antibodies against GLUT1 (1:2000, Novus Biological, Abingdon, U.K.), PARP (1:1000, Cell Signaling, Danvers, MA), MCT4 (1:1000, Santa Cruz, Dallas, TX), LC3 (1:1000, Cell Signaling), actin (1:500, Sigma-Aldrich), Cyclin D1 (1:1000, Cell Signaling), Beclin1 (1:1000, Abcam) and α-tubulin (1:4000, Sigma-Aldrich) followed by the appropriate secondary HRP-conjugated antibody (Amersham-Pharmacia, Amersham, U.K.). The signal was detected by chemiluminescence with SuperSignal kit (Life Technologies).

### RNA extraction, reverse transcription and qRT-PCR

Total RNA was extracted following the TRIzol protocol [17]. cDNA was synthesized from 0.5–1 μg of total RNA using the Superscript II reverse transcriptase (Life Technologies). Each sample was run in triplicate on ABI PRISM^®^ 7900HT Sequence Detection System (PE Biosystems, Foster City, CA). Results were analyzed using the comparative ΔΔCt method normalized to the housekeeping β_2_-microglobulin gene. Primer sequences are listed in Supplementary Table S2.

### Statistical analysis

Continuous variables were summarized with mean, standard deviation and median; categorical variables were reported as counts and percentages. Comparisons between mean values were performed by two-tail Student's *t* test. The χ^2^ test was used for the association between *in vitro* glucose addiction and clinical platinum response. Correlations between variables were evaluated by the Rho of Pearson. Progression Free Survival (PFS) was calculated from the completion of first-line chemotherapy to the time of last follow-up visit or event. The survival probability was estimated by means of the Kaplan-Meier method and heterogeneity in survival among strata was assessed through the log-rank test. The median PFS was reported along with 95% confidence intervals (CI). A *P*-value < 0.05 was considered as statistically significant. All data analyses were performed using the SAS statistical package (SAS, release 9.2; SAS Institute).

## SUPPLEMENTARY MATERIALS FIGURES AND TABLES


