# Supplementary material for: Resistance to glucose starvation as metabolic trait of platinum-resistant human epithelial ovarian cancer cells
Source: Oncotarget. 2016 Dec 23;8(4):6433–45. doi: 10.18632/oncotarget.14118 (PMC5351643; doi:10.18632/oncotarget.14118)
Supplement: Supplementary file 1 [file oncotarget-08-6433-s001.pdf]

Resistance to glucose starvation as metabolic trait of platinum-resistant human epithelial ovarian cancer cells

Supplementary Materials

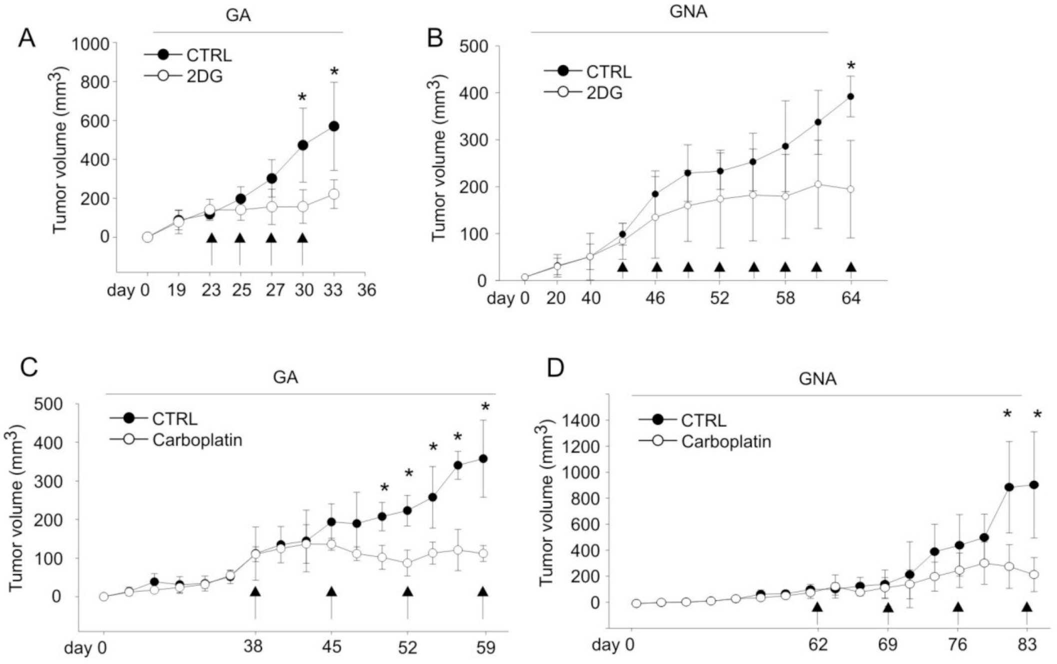

Supplementary Figure S1: Tumor cells from GA and GNA patients present different *in vivo* PLT-sensitivity. (**A–B**) Tumor growth curves in RAG-2$\gamma^{-/-}$ mice untreated (CTRL) and treated with the glucose analogue (2DG) after s.c. injection of cells from one representative GA (panel A) and GNA (panel B) PDX. When tumor volume reached 100 mm^3, the mice were randomized to receive the different treatments. The arrows indicate the days of 2DG administration; data show mean values (± SD) of six tumors/group. $*P < 0.05$. (**C–D**) Effect of carboplatin administration on the growth of tumors generated by s.c. injection of tumor xenotrasplants from one representative GA (panel C) and GNA (panel D) PDX into RAG-2$\gamma^{-/-}$ mice. It should be noted that GA/GNA cells used in this experiment were obtained from different PDX compared with those used to perform experiments shown in panels A and B. When tumor volume reached 100 mm^3, the mice were randomized to receive carboplatin or saline solution as a control (CTRL). The arrows indicate the days of drug administration; data show mean values (± SD) of six tumors/group. $*P < 0.05$.

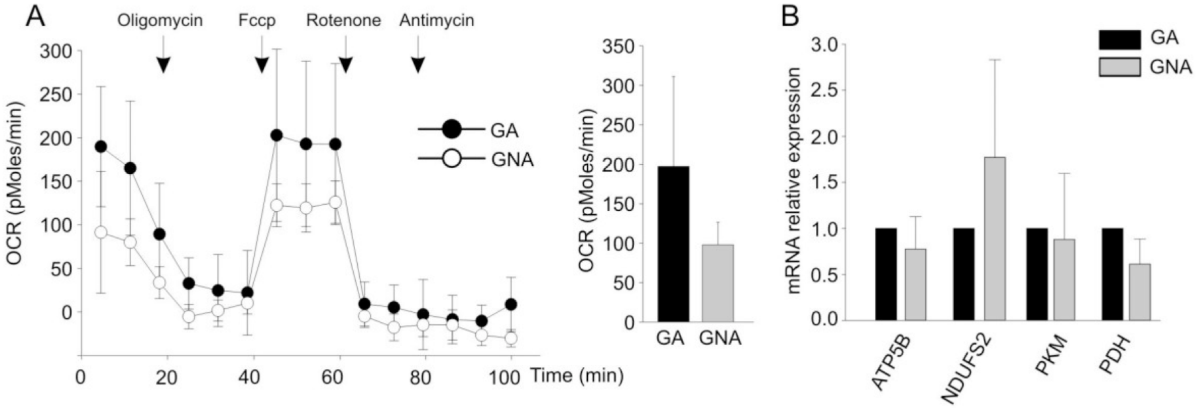

Supplementary Figure S2: Tumor cells from GA and GNA patients do not differ in terms of oxygen consumption. (**A**) Analysis of oxygen consumption rate (OCR) in CD45^{neg}/CD44^{pos} ascitic effusion cells from GA and GNA patients. Mitochondrial inhibitors were added as indicated. One representative experiment is shown on the left; the right histogram shows mean values (± SD) of the basal OCR in 6 GA and 6 GNA patients. (**B**) qRT-PCR analysis of key genes involved into mitochondrial activity. Bars showed mean values (± SD) of 6 GA versus 6 GNA samples.

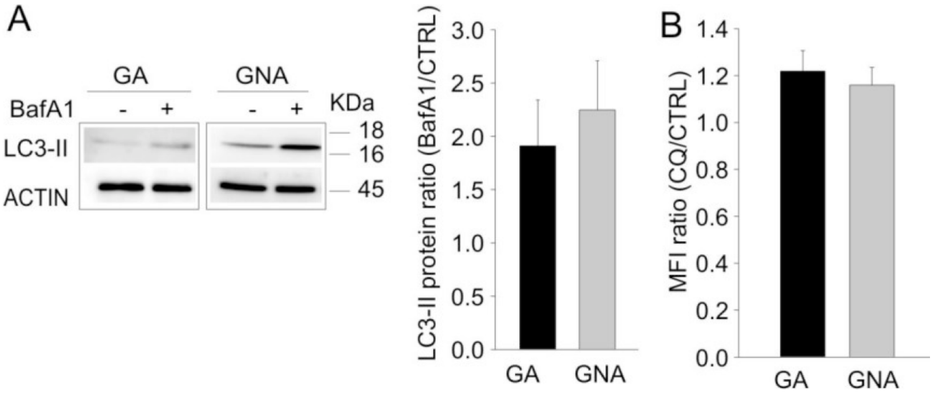

Supplementary Figure S3: GA and GNA samples present comparable autophagic flux. (**A**) WB analysis of LC3-II protein expression in CD45^{neg}/CD44^{pos} tumor cells from GA or GNA patients treated for 2 h with bafilomycin A1 (BafA1, 100 nM). One representative experiment is shown on the left; the right histogram depicts mean values (± SD) of 4 GA vs 4 GNA samples. Signal intensities were normalized against the actin signal. Expression ratios were calculated by dividing normalized signal intensity values obtained for untreated GA or GNA cells. (**B**) Flow cytometry analysis of autophagic flux activation in CD45^{neg}/CD44^{pos} cells after overnight chloroquine (CQ, 50 μM) treatment. Data show the mean values (± SD) of 4 GA and 4 GNA samples, expressed as mean fluorescence intensity (MFI) ratio between MFI values of CQ-treated and untreated (CTRL) cells.

Supplementary Table S1: The GA/GNA phenotype is maintained after xenotransplantation[a]

Patient ID	*Primary sample*		*Xenotransplant*	
	% cell viability	*GA/GNA phenotype*	*% cell viability*	*GA/GNA phenotype*
**#14**	1.8	GA	5.37	GA
**#24**	7.6	GA	12.3	GA
**#29**	11.2	GA	8.9	GA
**#36**	10.6	GA	4.31	GA
**#39**	5.7	GA	3.5	GA
**#49**	2.39	GA	2.92	GA
**#49bis**	6.35	GA	2.79	GA
**#52**	1.41	GA	9.21	GA
**#57**	0.37	GA	0.15	GA
**#60**	8.1	GA	11.7	GA
**#62**	4.9	GA	9.47	GA
**#70**	2.29	GA	5.4	GA
**#74**	6.76	GA	7.9	GA
**#79**	10.9	GA	6.01	GA
**#15**	45	GNA	53.4	GNA
**#17**	59.8	GNA	20.4	GNA
**#32**	66.5	GNA	82.0	GNA
**#37**	19.6	GNA	24.6	GNA
**#41**	43.1	GNA	36.4	GNA
**#44**	21.8	GNA	35.2	GNA
**#46**	18.6	GNA	13.6	GNA
**#50**	28.7	GNA	32.4	GNA
**#53**	46.2	GNA	21.1	GNA
**#54**	13.2	GNA	22.7	GNA
**#58**	75.6	GNA	80.1	GNA
**#69**	41.5	GNA	24.6	GNA
**#82**	21.2	GNA	47.2	GNA

a) EOC patients were categorized as GA or GNA according to the viability of their tumor cells after 14-day in vitro glucose starvation as detailed in Figure 1A (primary sample). The xenotransplants were generated by orthotopic injection of EOC tumor cells into SCID mice; the resulting tumor cells isolated from ascitic effusion were then assayed for their GA/GNA phenotype as above.

Supplementary Table S2: qRT-PCR primer sequences

Gene Name	Forward	Reverse
ABCG2	5'-CTCTTCGGCTTGCAACAACT-3'	5'-TTCTCCTCCAGACACACCAC-3'
ALDH1A	5'-CCCGTTGGTTATGCTCATTT-3'	5'-TGCTCTGCTGGTTTGACAAC-3'
ATP5B	5'-ATGACTTGACTGACCCTGCC-3'	5'-GGATAGATGCCCAGCTCAGC-3'
β2micro	5'-TCTCTCTTTCTGGCCTGGAG-3'	5'-TCTCTGCTGGATGACGTGAG-3'
CYCLIN A	5'-GGACCTTCACCAGACCTACC-3'	5'-AGTGTCTCTGGTGGGTTGAG-3'
CYCLIN B	5'-ACTGCCTGCTCTCACATCTT-3'	5'-TGTTGCAGGCAGGACAGATA-3'
CYCLIN D	5'-GGCGGAGGAGAACAAACAGA-3'	5'-GGAGGGCGGATTGGAAATGA-3'
CYCLIN E	5'-GTGGATGGCATCAAACAGGG-3’	5'-GCACCTTCCATAGCAGCATC-3'
GAPDH	5'-GAAGGTGAAGGTCGGAGT-3'	5'-CATGGGTGGAATCATATTGGAA-3'
GLUT-1	5'-GATGATGCGGGAGAAGAAGG-3'	5'-AAGACAGCGTTGATGCCAGAC-3'
HKII	5'-GAAGATGCTGCCCACCTTTG-3'	5'-CACCCAAAGCACACGGAAGT-3'
LDH-A	5'-GATTCAGCCCGATTCCGTTAC-3'	5'-ACTCCATACAGGCACACTGG-3'
LDH-B	5'-TGGGTGTTGGACAAGTTGGT-3'	5'-AGCAAGTTCATCAGCCAGAGA-3'
MCT-4	5'-GGCCGTCAGTGTCTTCTTCA-3'	5'-GACCTGTCCCGTAGAGCATG-3'
MRP1	5'-CAAGGTGGATGCGAATGAGG-3'	5'-TGAGGAAGTAGGGCCCAAAG-3'
MRP2	5'-GGACACATCTGCCATTCGAC-3'	5'-CCAGGTTCACATCTCGGACT-3'
NDUFS2	5'-GCAAGATCAAGGCTCCTGGT-3'	5'-GCAACGACATCTGCCAACAT-3'
PDH	5'-GACTGTACGCCGAATGGAGT-3'	5'-CCGGTAGGCTGTGATGAGAT-3'
PFK	5'-ACTGACGCCTGTCGCTTATG-3'	5'-GAGCGGGTTAGGTCCCTTCT-3'
PKM	5'-CATTGATTCACCACCCATCA-3'	5'-AGACGAGCCACATTCATTCC-3'
